# Storage Stability of Antioxidant in Milk Products Fermented with Selected Kefir Grain Microflora

**DOI:** 10.3390/molecules26113307

**Published:** 2021-05-31

**Authors:** Agata Biadała, Noranizan Mohd Adzahan

**Affiliations:** 1 Department of Food Quality and Safety Management, Faculty of Food Science and Nutrition, Poznan University of Life Sciences, Wojska Polskiego 31, 60-624 Poznan, Poland; 2 Department of Food Technology, Faculty of Food Science and Technology, University Putra Malaysia, Serdang 43400, Selangor, Malaysia; noraadzahan@upm.edu.my

**Keywords:** goat milk, kefir grain microflora, antioxidant activity

## Abstract

The aim of the study was to assess the antioxidant potential of goat’s milk and whey from goat’s milk fermented with selected bacteria strains from kefir grain (*L. plantarum*, *L. fermentum*, *L. rhamnosus* and *L. acidophilus*) with regard to fermented cow’s milk with the same bacteria strains. The assessment of antioxidant potential was made by ABTS, DPPH, TPC and FRAP methods. The work also assessed metabolic activity of tested lactic acid bacteria using measurement of electrical impedance changes in the growing medium. The highest values describing the antioxidant potential were found for fermented milk by *L. acidophilus*. It was also found that the time of cooling storage causes significantly increasing the antioxidant potential of most analyzed samples. Metabolic activity of tested lactic acid bacteria was the highest for cow’s milk. The course of curves for goat’s milk and whey from goat’s milk was similar, which confirms the differences between cow and goat milk.

## 1. Introduction

Consumers around the world are increasingly seeking products with beneficial effects on health. Goat milk and its fermented products meet this demand. This is attributed by its high nutritional value and numerous pro-health properties [1]. In addition, goat milk and its fermented products are known to have lower allergenic potential and better digestibility [2]. The exceptional health-promoting benefits is related to the composition of fatty acids, smaller fat globule size, protein quality and abundance in minerals and vitamins [3,4].

Antioxidants are chemical compounds that inhibit the production of free radicals, resulting in a limitation of cell damage. As indicated by the name, antioxidants prevent oxidation and neutralizes free radicals by giving up some of their own electrons. Fermented milk products are characterized by their antioxidant potential after processing [5]. The antioxidant properties may vary depending on the duration of storage and fermentation conditions. Kefir from goat’s milk has the ability to scavenge free radicals, characterized by an increase in chelating activity of iron ions (Fe^2+^) and a decrease in phenolic content during fermentation [6]. The addition of the kefir grain results in an increase in antioxidant properties, making a new kefir range with pro-health properties for consumers [7,8,9]. There are reports on the antioxidant properties of kefirs produced from goat’s milk using commercially available starter cultures. However, data on specific microorganism in the kefir grain microflora with the greatest impact on the antioxidant property of kefir from goat milk is scarce. The antioxidant capacity of milk and its products is primarily due to the presence of amino acids containing sulfur, particularly cysteine, as well as vitamins A, vitamin E, carotenoid, zinc, selenium and enzymatic systems to which superoxide, catalase and gluthionase peroxidase belong. In this aspect, goat contains more when compared to the popular cow milk [4,8].

Whether antioxidant properties in fermented milk products can be used as direct indicator to proteolytic processes in whey proteins are yet to be confirmed. Thus, the aim of this study is to check if the fermentation with selected, single strains of microorganisms from kefir grain microflora can influenced on antioxidant activity of fermented goat milk and if are there significant changes of examined activity during cooling storage. An understanding of this matter would allow manipulation of processes involved in milk production as to produce products with the highest antioxidant properties. This knowledge could also be used to design starter cultures for goat milk products with enhanced health-promoting properties.

## 2. Results and Discussion

### 2.1. Analysis of Metabolic Activity of Selected Lactic Acid Bacteria

**Figure 1:**
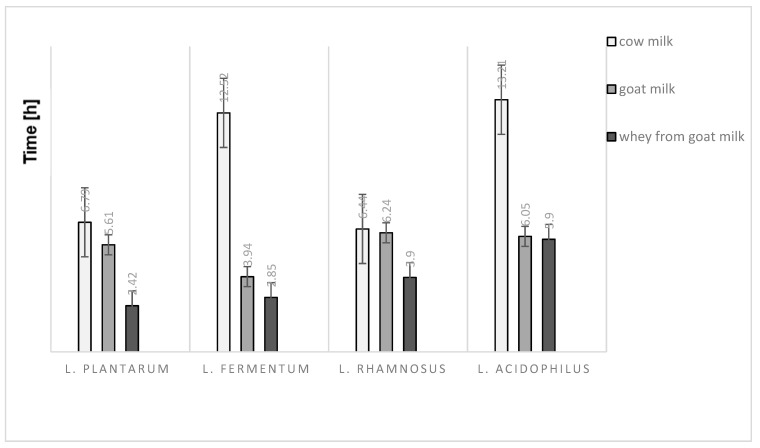
The detection time of impedance changes at 5% in the different kind of milk and milk fraction.

Various media resulted in varying degree of electrical impedence but with similar patterns between the different samples tested. Regardless of lactic acid bacteria strains used, whey from goat milk seems to have the shortest time for impedence change. On the contrary, the time to detect an impedence change was longest for cow milk which is a reference sample in this experiment (Figure 1). The time taken for impedence changes in cow milk fermented using *L. fermentum* and *L. acidophilus*, were at least double the time recorded for other samples.

**Figure 2:**
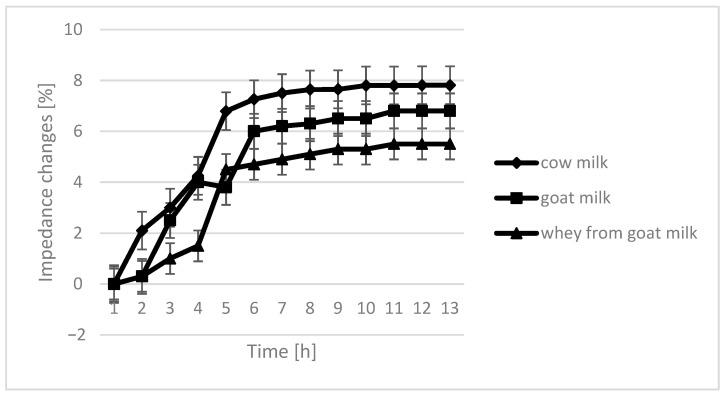
Impedance changes in different medium caused by *L. plantarum* growth.

**Figure 3:**
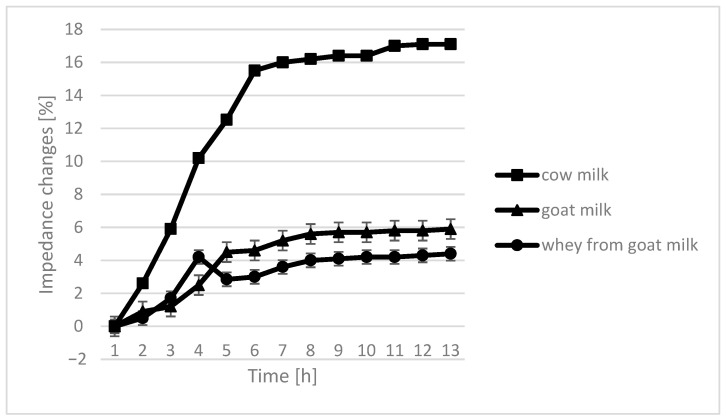
Impedance changes in different medium caused by *L. fermentum* growth.

**Figure 4:**
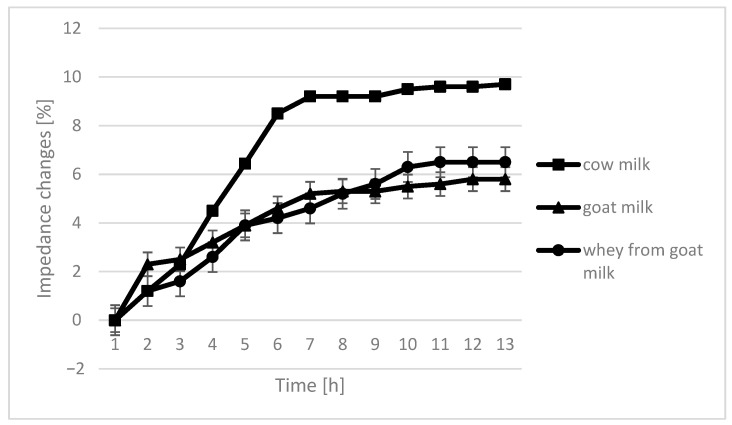
Impedance changes in different medium caused by *L. rhamnosus* growth.

**Figure 5:**
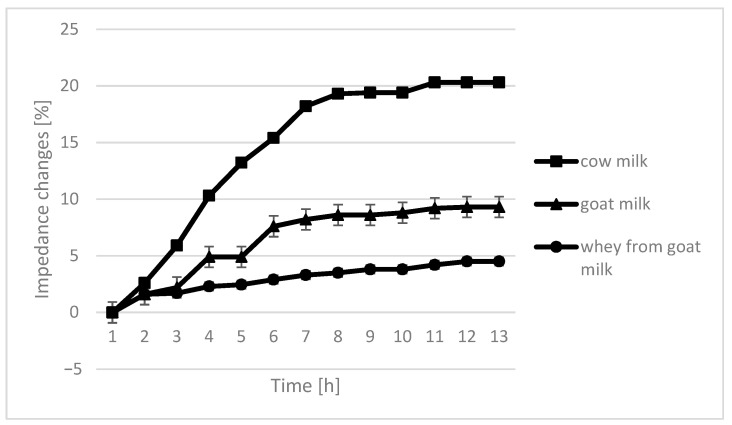
Impedance changes in different medium caused by *L. acidophilus* growth.

The course of the curves for impedance change in individual growth medium was similar, however, with cow milk having the highest percentage of impedence change (Figure 2, Figure 3, Figure 4 and Figure 5).

The only instance in which no significant differences are seen in the course of curves are samples fermented by *L. plantarum*. In the case of cow milk, the dynamics of impedance changes are the largest. In the case of goat’s milk and whey from goat milk, metabolic changes take care of similar intensity.

Monitoring of impedance changes can be used not only for checking which microorganisms is the most appropriate for each type of medium [10,11] but also to predict in which product of fermentation the biggest amount of metabolic remains are present [11].

### 2.2. Antioxidant Potential and its Changes During Cooling Storage

The antioxidant activity for the three milk products tested differed significantly (*p* < 0.05) and this was demonstrated by both the DPPH and ABTS methods. The highest antioxidant properties were recorded in whey of goat milk that was fermented by *L. acidophilus* after 14 days of refrigerated storage (Table 1 and Table 2).

Obtaining higher values in the ABTS method than DPPH can be the result of ability to capture ABTS redical cations, through some of the milk components: cysteine, glutathione or sulfhydryl proteins [8]. Similar results were reported by Yilmaz-Ersan et al. [8]. The highest FRAP values were for product obtained for fermented goat’s milk (Table 3).

In each of the raw materials that have been used to produce fermented samples, there are both hydropobic and hydrophilic antioxidant components. In the hydrophobic phase vitamin E and carotenoids play the primary role in the antioxidant potential, while in the water-soluble phase, ascorbic acid (vitamin C) is the primary antioxidant. According to Yilmaz-Ersan et al. [8] differences in antioxidant capacity between goat milk, cow milk or whey from goat milk may result from their relative fat content, proteins, vitamins, minerals, phenolic compounds, antioxidant enzymes and compositions of fatty acids. It may be possible that the content of antioxidant components in the raw material may affect the antioxidant potential of their fermented products [12,13].

The lowest antioxidant capacity against the DPPH radical which was recorded for fermented samples from goat milk was 1.66 mg TE/100 mL. This value corresponds to a fermented *L. plantarum*. Fermentation of goat milk with *L. fermentum* and *L. acidophilus* and holding under refrigeration for 14 days led to the highest antioxidant potential as determined by the DPPH method. Refrigeration significantly influenced the increase of the antioxidant potential of the analysed samples by DPPH method in goat milk fermented by *L. plantarum*, *L. acidophilus* and *L. rhamonsus*. In the case of whey, a significant increase in potential was found after 14 days of storage in fermented sample by *L. acidophilus* and *L. fermentum*.

Based on monitoring of the antioxidant capacity for the ABTS radical cation for fermented milk prepared from whey from goat milk, it was found that the highest value was recorded for milk fermented by *L. acidophilus* (Table 1). In turn, the lowest antioxidant potential corresponds to a sample of milk just after incubation by *L. acidophilus*. A significant increase in antioxidant potential was recorded for goat milk and whey samples which were fermented by *L. fermentum*, *L. rhamnosus* and *L. acidophilus* after refrigerated storage. The lowest antioxidant potential in relation to the ABTS radical cation monitoring, using goat milk as a raw material, corresponded to a milk fermented by *L. acidophilus*.

The Folin-Ciocalteu assay for total phenolic content (TPC) determination is a commonly used method for identifying the presence of phenolics, the molecules with the highest potential to neutralize free radicals. This method has some limitations resulting from fact that this reagent reacts with all other reducing agents that can be found in milk like peptides, amino acids, vitamins and minerals [14]. The lowest average TPC value was found for fermented cow’s milk (Table 4). There were large discrepancies in the TPC results for all types of milk fermented by lactic acid bacteria strains. This was probably caused by inhibition of enzymes from lactic acid bacteria. TPC values obtained were within a 49.76 to 98.66 mg GAE/100 mL range. The lowest values were seen for the cow’s milk samples fermented with the analyzed strains and the highest in a sample from goat’s milk. Cooled storage significantly increased the antioxidant potential measured by TPC with the exception of samples of whey from goat’s milk fermented by *L. plantarum* and *L. rhamnosus*. This is a result of formation by the microorganisms of peptides and amino acids by hydrolysis which then give a positive reaction with Folin reagent [15,16].

FRAP values significantly increased during the cold storage of each analyzed sample. The lowest value was seen for whey from goat’s milk fermented by *L. fermentum* immediately after the incubation process, while the highest value was for goat’s milk fermented by *L. plantarum*.

Kefir is characterized by higher antioxidant potential than milk. In the research conducted by Liu et al. [17], increased milk activity was demonstrated by fermented milk with kefir grains in the aspect of capturing DPPH radicals compared to non-fermented milk. Therefore, the use of individual strains of microorganisms included in the kefir grain microflora, for milk fermentation, positively affected the antioxidant quality of the resulting beverage. The microorganism strain used for the fermentation very much determines the value of such a beverage. The conditions for lactic acid bacteria growth are strict and require a carbon source, nitrogen source, inorganic salts and prebiotics. The nutrients are not only necessary to grow the bacteria but also to promote the production of proteinase. Research by Shu et al. suggested that casein peptone, glucose and calcium lactate had significant effects on the antioxidant peptides production in fermented goat’s milk [18,19].

The antioxidant capacities of refrigerated fermented samples were significantly different (*p* < 0.05) during the 14 days of storage as shown by the ABTS, DPPH, TPC and FRAP results. Fermented samples from whey from goat’s milk, which were analyzed after production, are characterized by a significantly lower (*p* < 0.05) antioxidant potential and a significantly lower data spread. Yilmaz-Ersan et al. [8] studied the antioxidant activity of kefir from goat milk and found that immediately after the addition of kefir grain to the samples, there is an increase in the rate of capture of free DPPH radicals. After 8 h of fermentation, the greatest growth was noticed. The DPPH radical capturing activity increased with increasing storage time. The highest DPPH antioxidant performance was recorded after kefir storage for 21 days under cool conditions, and for the ABTS method after 14 days. It was also noticed that ABTS activity in kefir from goat’s milk changes during the fermentation process. ABTS antioxidant activity was detected between 14 and 21 days of storage under refrigerated conditions. These results also led them to conclude that the kefir antioxidant activity depends on the fermentation conditions and storage time. In another study it was reported that the instability of the antioxidant activity of kefir may be related to the type of milk used, chemical composition, processing conditions, type and quantity of starter culture used, population of microorganisms contained in the kefir grain microflora and a kind of enzymes contained in the starter culture [8].

According to Zhang et al. [17] after fermentation of goat milk, the DPPH radical capturing activity increased, while in the case of cow’s milk, such changes were not observed. This phenomenon could result from a lower casein content in goat’s milk, and therefore it was easier to hydrolyse by proteolytic enzymes. In addition, the peptides formed as a result of the fermentation of milk proteins have antioxidant properties too. Levels of amino acids such as histidine, tryptophan, tyrosine, phenylalanine or proline correlate with antioxidant properties. A higher level of some amino acids in goat’s milk leads to an increase in the DPPH radical capturing activity compared to cow’s milk. Alyaqoubi et al. [20] compared the antioxidant properties of pasteurized and unpasteurized goat milk. They showed that the pasteurization process significantly (*p* < 0.05) reduced the antioxidant activity. This could have resulted from a reduction in the content of flavonoids with antioxidant properties and the high sensitivity to albumin to thermal treatment. The increase in antioxidant potential during the refrigerated storage is caused by the proteolysis process, which occurs in the product and increases the contents of organic acids [7].

## 3. Materials and Methods

### 3.1. Raw Material

The test material used was milk from cow and goat as well as whey from goat milk. Both types of milk were obtained from ecological farms in the Wielkopolska region of Poland. Both types of milk were pasteurized at 93°C for 5 min. Next milk was poured to a glass bottle (DURAN^®^ Protect, DURAN Group GmbH, Wertheim), and cooled to a temperature of 30 °C ± 1. The whey component was obtained via a microfiltration process.

### 3.2. Whey Separation

Membrane filtration was conducted using the Isolux membranes with modified filtering layers (TAMI, Nyons, France) characterized by 1.4 μm and 0.2 μm cut-off values. The above process was carried out at 20 ± 2 °C. The initial pressure was 6 atm and was reduced to 3 atm during the process [16]. The transmembrane preassure was 0.41 bar and the velocity through the membrane was 2 m/s.

### 3.3. Inoculation and Fermentation

Microorganisms used in the experiment (*L. plantarum* PCM 1386, *L. acidophilus* PCM 2499, *L. fermentum* PCM 491, *L. rhamnosus* PCM 2677) was obtained from the Polish Collection of Microorganisms, Institute of Immunology and Experimental Therapy Polish Academy of Sciences (Wrocław, Poland) and Deutsche Sammlung von Mikroorganismen und Zellkulturen GmbH (DSM); Leibniz Institute, Braunschweig, Germany). Bacteria innocula were preapared in MRS medium. Each strain was transformed into MRS medium and incubated in 30 ± 1 °C for 48 h. Fermented samples were prepared through material inoculation with a single strain of the selected kefir grain microflora (10^8^ cfu/mL) and then carrying out the incubation process at 30 ± 1 °C for 24 h. After the fermentation process, the sample was cooled to 4 ± 1 °C and kept under cooled conditions for 14 days.

### 3.4. Evaluation of Bacterial Metabolic Activity

The evaluation of bacterial metabolic activity was performed by a direct method by registering impedance changes directly in the growth medium using a BacTrac 4100 Automatic Microorganism Growth Analyzer [21].

### 3.5. Determination of Total Phenolic Content

The total phenolic content (TPC) was determined by the Folin–Ciocalteu method as described by Sahin et al. [22,23]. Fermented samples (100 μL) were mixed with 2 mL of 2% sodium carbonate solution. After 3 min, 100 μL of Follin-–Ciocalteu reagent was added. The absorbance was measured using a UV-Vis spectrophotometer (Varian Cary, Walnut Creek, CA, USA at 750 nm against a reagent blank after 30 min at room temperature. A standard calibration curve was plotted using different concentrations of gallic acid and TPC was expressed in milligrams of gallic acid equivalents (GAE) per 100 mL of sample.

### 3.6. Determination of Antioxidant Capacity

The antioxidant capacity was studied by evaluating the free-radical scavenging effect on DPPH radicals. For the DPPH assay, the method of Aguilar-Toalá et al. [6] was used with some modifications. The DPPH model solution needed for analysis was prepared from 1 mL of 1,1-diphenyl-2-picrylhydrazyl and 80 mL of methanol. The absorbance was measured at 515 nm using a spectrophotometer (Cary 60 UV-Vis, Agilent, Walnut Creek, CA, USA). Fermented milk samples (50 μL), 2.5 mL of the prepared DPPH solution, and 1.25 mL of chloroform were placed in a test tube and left protected from light and at room temperature for 15 min, followed by centrifugation for 5 min at 8000 rpm. The resulting supernatant was poured off and the absorbance measured using a Cary 60 UV-Vis spectrophotometer at the wavelength of λ = 515 nm against a mixture of methanol and chloroform in a 2:1 ratio. The measurement was carried out via a fiber optic probe by immersing it in the tested sample.

The ABTS model solution needed for analysis was made from 1 mL of 2,2′-azino-bis-3-ethylbenzothiazoline-6-sulfonic acid and 80 mL of methanol added to a beaker [16]. The absorbance of the resulting solution was brought to 0.700 ± 0.002 by monitoring with the Cary 60 UV-Vis spectrophotometer, at a wavelength of 734 nm. Fermented milk (30 μL) and 3 mL of the previously prepared ABTS solution were added to the tube. Two repetitions of a blank test were also done. Two glass tubes were loaded with 30 μL of methanol and 3 mL of ABTS solution. Samples were stored for 10 min in a dark room at room temperature. Then samples were centrifuged at 8000 rpm for 5 min. The resulting supernatant was decanted and the absorbance was measured on the Cary 60 UV-VIS spectrophotometer at the wavelength of λ = 734 nm against methanol. The measurement was carried out via a fiber optic probe immersed in the tested sample.

### 3.7. Ferric Reducing Antioxidant Power (FRAP) Assay

The ferric reducing antioxidant power (FRAP) assay was performed using the method of Benzie and Strain [24] and a UV-Vis spectrophotometer (Varian Cary 50) with slight modifications [15,16]. The method, using iron as a redox sensor, is based on the reduction of the Fe^3+–^TPTZ (ferric tripyridyltriazine) complex to the corresponding Fe^2–^TPTZ complex at low pH in the presence of antioxidants. This reduction results in a blue color, which can be monitored by measuring the absorption change at 593 nm. The working FRAP reagent was prepared by mixing 10 volumes of 1.0 M acetate buffer, pH 3.6, with 1 volume of 10 mM TPTZ in 40 mM HCl and with 1 volume of 20 mM FeCl_3_. In a test tube, 0.25 mL of sample was mixed with 2.75 mL of the FRAP reagent and incubated at 37°C for 30 min. The solution was then centrifuged at 1050× *g* for 10 min. The supernatant (0.5 mL) was mixed with 0.5 mL of distilled water and 0.1 mL of 0.1% (wt/vol) FeCl_3_. Absorbance was measured after 8 min. A standard curve was prepared using different concentrations of FeSO_4_ × 7H_2_O (100–1,000 μM). The antioxidant capacity was determined from a standard curve generated using FeSO_4_. The FRAP results were expressed as moles of FeSO_4_ per kilogram of fresh weight. The antioxidant efficiency of the sample solution was calculated with reference to the standard curve given by a Fe^2+^ solution of known concentration. The results were expressed as milligrams of TE per 100 mL of sample.

### 3.8. Statistical Analyses

All data were presented as the mean of the triplicate determinations. The data were analyzed using the Statistica 13.3 (Statsoft Inc., Kraków, Poland) software. In order to compare the differences between data characterized by a normal distribution, a Student’s *t*-test was performed, while in the case of data not having a normal distribution, a Wilcoxon para sequence test was used. A comparative interpretation of antioxidant potential for fermented beverages during their production and after 14-day storage under refrigeration conditions was made using a single-factor analysis of variance (ANOVA). All analyses were carried out at a significance level of 5% (P ≤ 0.05).

## 4. Conclusions

The greatest impedance changes were observed during the first 8–10 h of incubation. After this time, metabolic activity entered a stationary phase. The highest changes and values describing antioxidant activity were observed during the fermentation process. The storage time resulted in a significant increase of the antioxidant activity for the tested samples. This is caused by the proteolytic activity of lactic acid bacteria which was not halted despite the refrigerated storage temperatures. It was found that the storage time does not inhibit the changes leading to increasing the antioxidant properties of products and only clearly slows it down.

## Figures and Tables

**Table 1:** Changes in antioxidant capacity (ABTS) in mg of TE/100 mL.

		*L. plantarum*	*L. acidophilus*	*L. fermentum*	*L. rhamnosus*
**Cow’s mik**	after incubation	25.41 ± 0.025 ^A^	20.54 ± 0.006 ^A^	11.93 ± 0.027 ^A^	21.43 ± 0.018 ^A^
	after 14 days storage	26.13 ± 0.014 ^A^	24.93 ± 0.014 ^B^	29.74 ± 0.014 ^B^	33.68 ± 0.019 ^B^
**Goat’s milk**	after incubation	24.33 ± 0.009 ^A^	11.93 ± 0.005 ^A^	15.41 ± 0.002 ^A^	21.16 ± 0.028 ^A^
	after 14 days storage	26.94 ± 0.011 ^A^	19.81 ± 0.018 ^B^	36.44 ± 0.017 ^B^	34.82 ± 0.031 ^B^
**Whey from goat’s milk**	after incubation	24.14 ± 0.025 ^A^	30.12 ± 0.027 ^A^	12.83 ± 0.019 ^A^	42.19 ± 0.005 ^A^
	after 14 days storage	27.33 ± 0.019 ^A^	63.43 ± 0.014 ^B^	35.40 ± 0.027 ^B^	42.46 ± 0.017 ^A^

* means with different superscripts within same column for each kind of milk are significantly different (*p* < 0.05).

**Table 2:** Changes in antioxidant capacity (DDPH) in mg of TE/100 mL.

		*L. plantarum*	*L. acidophilus*	*L. fermentum*	*L. rhamnosus*
**Cow’s mik**	after incubation	1.97 ± 0.017 ^A^	1.89 ± 0.014 ^A^	1.69 ± 0.019 ^A^	1.96 ± 0.027 ^A^
	after 14 days storage	2.95 ± 0.009 ^B^	3.89 ± 0.008 ^B^	3.74 ± 0.008 ^B^	3.95 ± 0.027 ^B^
**Goat’s milk**	after incubation	1.66 ± 0.002 ^A^	2.66 ± 0.021 ^A^	3.65 ± 0.034 ^A^	2.13 ± 0.011 ^A^
	after 14 days storage	3.57 ± 0.011 ^B^	4.17 ± 0.007 ^B^	4.17 ± 0.016 ^A^	3.33 ± 0.021 ^B^
**Whey from goat’s milk**	after incubation	1.87 ± 0.007 ^A^	1.86 ± 0.017 ^A^	1.37 ± 0.014 ^A^	1.46 ± 0.006 ^A^
	after 14 days storage	2.01 ± 0.008 ^A^	4.87 ± 0.006 ^B^	3.67 ± 0.021 ^B^	1.06 ± 0.029 ^A^

* means with different superscripts within same column for each kind of milk are significantly different (*p* < 0.05).

**Table 3:** Changes in antioxidant capacity (FRAP) in mg of TE/100 mL.

		*L. plantarum*	*L. acidophilus*	*L. fermentum*	*L. rhamnosus*
**Cow’s mik**	after incubation	36.97 ± 1.064 ^A^	25.71 ± 0.098 ^A^	26.75 ± 0.987 ^A^	29.77 ± 0.961 ^A^
	after 14 days storage	56.98 ± 2.014 ^B^	56.19 ± 0,995 ^B^	53.42 ± 1.547 ^B^	56.79 ± 2.004 ^B^
**Goat’s milk**	after incubation	40.29 ± 0.964 ^A^	32.58 ± 1.201 ^A^	21.12 ± 0.995 ^A^	22.21 ± 1.204 ^A^
	after 14 days storage	69.76 ± 1.954 ^B^	59.75 ± 2.036 ^B^	59.76 ± 1.364 ^B^	62.73 ± 0.964 ^B^
**Whey from goat’s milk**	after incubation	41.12 ± 0.993 ^A^	29.49 ± 0.987 ^A^	19.77 ± 0.987 ^A^	26.78 ± 1.987 ^A^
	after 14 days storage	65.45 ± 1.982 ^B^	55.47 ± 1.201 ^B^	49.36 ± 0.996 ^B^	59.19 ± 1.247 ^B^

* means with different superscripts within same column for each kind of milk are significantly different (*p* < 0.05).

**Table 4:** Total phenolic content after incubation and after 14 days of cooling storage (mg of GAE/100 mL).

		*L. plantarum*	*L. acidophilus*	*L. fermentum*	*L. rhamnosus*
**Cow’s mik**	after incubation	69.77 ± 1.005 ^A^	62.47 ± 1.54 ^A^	64.97 ± 0.987 ^A^	63.95 ± 0.843 ^A^
	after 14 days storage	78.63 ± 0.991 ^B^	78.55 ± 0.992 ^B^	73.13 ± 1.247 ^B^	74.38 ± 0.981 ^B^
**Goat’s milk**	after incubation	69.76 ± 1.230 ^A^	56.95 ± 2.007 ^A^	65.15 ± 0.998 ^A^	49.76 ± 1.021 ^A^
	after 14 days storage	98.66 ± 1.025 ^B^	79.47 ± 1.364 ^B^	89.16 ± 0.947 ^B^	62.17 ± 0.247 ^B^
**Whey from goat’s milk**	after incubation	65.44 ± 1.998 ^A^	55.47 ± 0.996 ^A^	55.77 ± 2.001 ^A^	51.26 ± 1.297 ^A^
	after 14 days storage	65.22 ± 2.004 ^A^	79.01 ± 1.221 ^B^	76.17 ± 1.257 ^B^	59.74 ± 0.187 ^A^

* means with different superscripts within same column for each kind of milk are significantly different (*p* < 0.05).
